# Sex Differences in Suicide Attempts: A Cross-Sectional Study in Patients with First-Episode and Drug-Naïve Major Depression Disorder

**DOI:** 10.1155/2024/5391546

**Published:** 2024-07-26

**Authors:** Yingzhao Zhu, Jun Zhang, Junjun Liu, Fengnan Jia, Zhe Li, Xueli Zhao, Chuanwei Li, Hanxu Deng, Yue Zhou, Xingzhi Xia, Ruchang Yang, Xiangdong Du, Xiangyang Zhang

**Affiliations:** ^1^Medical College of Soochow University, Suzhou, China; ^2^Suzhou Guangji Hospital, The Affiliated Guangji Hospital of Soochow University, Suzhou, China; ^3^Nanjing Meishan Hospital, Nanjing, China; ^4^Xuzhou Medical University, Xuzhou, China; ^5^Affiliated Mental Health Center of Anhui Medical University, Hefei Fourth People's Hospital, Anhui Mental Health Center, Hefei, China

## Abstract

**Materials and Methods:**

A cross-sectional study was conducted on the FEDN patients with MDD, and 1,718 patients' demography information and clinical data were collected. The Hamilton Depression Scale (HAMD), Hamilton Anxiety Scale (HAMA), and Positive and Negative Syndrome Scale (PANSS) were used to evaluate symptoms of depression, anxiety, and psychotic symptoms, respectively. Thyroid hormones, lipid profile, and fasting blood glucose (FBG) were measured. A history of suicide attempt was verified based on medical records and interviews with patients and their families. A 2 × 2 ANOVA was used to compare the clinical parameters of MDD patients in the suicide attempts subgroup and the sex subgroup, as well as whether there is an interaction between these two subgroups. Univariate analysis and multivariate binary logistic regression analyses were used to assess factors associated with suicide attempts.

**Results:**

There was no sex difference in rates of suicide attempt among FEND patients with MDD (male: 19.0% vs. female: 20.7%, *χ*^2^ = 0.663, *p*=0.416). Compared to males and females without suicide attempts, those with suicide attempts had higher levels of LDL-C and lower levels of HDL-C. There was a statistically significant difference in the course of disease, educational level, and TG in the female group but not in the male group. In both male and female patients, Lg (TPOAb) and severe anxiety symptoms were found to be positively correlated with suicide attempts. In addition, in male patients, suicide attempts were positively correlated with TC and FBG, while negatively correlated with body mass index. In female patients, there was a positive correlation between the severity of depression and elevated systolic blood pressure with suicide attempts (all *p* < 0.05).

**Conclusion:**

Our study showed that there is no sex difference in the prevalence of suicide attempts in FEDN patients with MDD and there are differences in factors related to suicide attempts between male and female MDD patients.

## 1. Introduction

Major depression disorder (MDD) is a highly prevalent and severe mental illness, manifesting as persistent low mood, fatigue, anhedonia, and a heightened risk of suicidal ideation and behavior. Globally, it is estimated to impact ~350 million individuals, presenting a significant risk of recurrence and constituting a substantial public health burden [1, 2, 3, 4]. Recent statistics from the United States [5] indicated that, between 2013 and 2016, 8.1% of adults aged 20 and over experienced episodes of depression over a 2-week period. Comparable data from China suggested a lifetime prevalence of MDD at 3.4%, with a 12-month prevalence rate of 2.1% [6], highlighting the universal challenge posed by depression across diverse populations. A systematic review and meta-analysis further revealed a growing trend in the prevalence of depression, suggesting an increasing likelihood of developing the condition over time [7]. The World Health Organization (WHO) [8] has acknowledged the critical impact of depression, ranking it as the third leading cause of global disease burden and projecting it to become the foremost cause by 2030 [8]. These findings suggested that the burden of depression is expected to increase further in the coming years. Therefore, there is an urgent need to raise awareness of depression, early recognition, and effective treatment.

Depression can lead to functional impairment in patients, and most seriously, it can lead to suicide [9, 10]. Studies have consistently demonstrated that individuals with severe depression are at an increased risk of mortality [11]. For example, a cross-border summary of data revealed that 9.2% of individuals experience suicidal ideation, 3.1% formulate plans for suicide, and 2.7% attempt suicide [12]. However, within populations diagnosed with major depressive disorder (MDD), the lifetime prevalence of suicide attempts has been reported to be as high as 31%, with an annual prevalence rate of 8% [13]. Therefore, factors related to suicide attempts in MDD have been extensively studied, including biological, psychological, and environmental factors [14]. For example, an earlier onset of depressive symptoms [15], clinical features like the severity of depressive pathology [16], comorbid physical illnesses [17], and substance abuse or dependence [18] are all considered to be associated with suicide attempts in MDD. Additionally, a history of previous suicide attempts [19] and a lack of physical activity [20] are also significant contributors to the risk profile for suicide attempts in this population.

In addition, MDD patients also exhibit sex differences in suicide attempts [21, 22]. For example, Olgiati et al. [23] reported that men have more severe suicidal attitudes and more suicide risk factors such as alcohol abuse and weight loss. On the contrary, studies have shown that female patients have a higher incidence of suicide attempts compared to male patients [24]. Moreover, a community-based meta-analysis has elucidated a positive association between depression and obesity, a link that is notably stronger in women [25]. Additionally, the severity of depression has been found to correlate positively with the risk of suicide attempts, a relationship that is also modulated by sex differences [16, 26].

However, research on sex differences related to suicide attempts in MDD patients is not comprehensive and controversial. Although previous research findings have confirmed significant sex differences in suicide attempts among MDD patients, more emphasis has been placed on demographic and clinical symptom differences, with less emphasis on clinical variables. Therefore, individuals with first-episode, drug-naïve (FEND) major depressive disorder (MDD) present a unique cohort for investigating sex disparities in suicide attempt rates among MDD patients, without being confounded by lifestyle changes and medication treatment. The purpose of this study is to examine sex differences in two key domains: (1) demographics and clinical presentation, as well as the incidence of suicide attempts; and (2) associated factors of suicide attempts among Chinese patients with MDD.

## 2. Materials and Methods

### 2.1. Subjects

A cross-sectional analysis was conducted, encompassing data from 1,718 patients who visited the First Affiliated Hospital of Shanxi Medical University from 2016 to 2018. This cohort comprised solely first-time and medically naïve MDD patients, with a sex distribution of 588 males and 1,130 females, based on biological sex. All diagnoses of MDD were made by two qualified psychiatrists, and their diagnoses were consistent. The enrolled patients need to meet the following conditions: (1) Han nationality, aged between 18 and 60 years old; (2) first-episode and drug-naïve depression patient; (3) meet the diagnostic criteria for MDD in the Structured Clinical Interview for DSM-IV [27]; and (4) the total score of the HAMD scale is ≥24. At the same time, it is necessary to exclude the following situations: (1) pregnancy or breastfeeding; (2) abuse or dependence on substances other than tobacco; (3) having severe personality disorders; (4) suffering from serious physical illnesses; (5) refusal to participate in research; (7) unable to accept interviews due to urgent clinical situations; and (8) other unspecified reasons.

This study was approved by the Ethics Committee of Shanxi Medical University's First Hospital (No. 2016-Y27), and all participants signed a written informed consent form.

### 2.2. Sociodemographic Information and Anthropometric Data

We used a self-made questionnaire to collect basic information about patients, such as sex, age, education level, marital status, age of onset, and course of disease. We also measured systolic and diastolic blood pressure (SBP and DBP) and calculated the patient's body mass index (BMI, calculated as weight/height^2^).

Suicide attempts are judged by the following question: “Have you ever suicide attempts in your life?” This question comes from a multicenter study by the World Health Organization/Europe [28]. People who replied “yes” were considered a suicidal attempter. We then asked them for more information about the number of suicide attempts, their methods, and the exact dates. If their answers were vague, we would get more information by interviewing their family or friends. According to statistics, a total of 346 patients with MDD had a suicide attempt before the first diagnosis, including 317 patients with one attempt, 26 patients with two attempts, two patients with three attempts, and one patient with four attempts. Furthermore, there were 111 patients had a suicide attempt 1 month before the first diagnosis, and 235 patients had suicide attempts within 2 weeks, including 43 who cut their wrists, 23 who hanged themselves, 31 who drowned themselves (including jumping into rivers, wells, lakes, and bridges), 14 who tried to jump off buildings (including jumping from heights), 20 who committed suicide by using gas (including charcoal), 54 who swallowed drugs (various drugs), and 22 who committed suicide by taking poison (including pesticides, rodenticides, and cockroach drugs), 11 suicided by crashing into a car, and other ways included drinking gasoline, jumping out of the car, high-voltage electricity, hitting the wall, etc.

### 2.3. Clinical Scale Evaluation

The Hamilton Depression 17-item scale (HAMD-17) [29] was used to assess the severity of depression in patients. This particular measurement method has been widely used in numerous studies and has proven to be effective in capturing changes in depression symptoms in patients. The HAMD-17 comprises a total of 17 items, with eight items scored on a 5-grade scale ranging from 0 (representing no symptoms) to 4 (representing extreme severity), and nine items scored on a 3-grade scale ranging from 0 (representing no symptoms) to 2 (representing severe symptoms). The HAMD-17 score ranges from 0 to 52, with higher scores indicating more severe symptoms. In this study, a HAMD score of ≥24 was identified as indicative of severe depressive symptoms.

The Hamilton Anxiety Scale (HAMA) [30] was employed to assess the severity of anxiety symptoms. The HAMA assesses two major categories of anxiety symptoms, namely physical anxiety and mental anxiety, consisting of a total of 14 items scored on a 5-grade scale ranging from 0 to 4. Patients with a HAMA score of 25 or higher are considered to have severe anxiety symptoms [31].

The positive scale scores in the Positive and Negative Syndrome Scale (PANSS) [32] were used to evaluate the psychotic symptoms of patients. The positive subscale, comprised of seven items, uses a 7-level rating scale ranging from 1 (indicating nonexistence of symptoms) to 7 (indicating extreme severity of symptoms). A score of ≥15 on the positive subscale is regarded as indicative of a psychiatric symptom.

All the psychiatrists participating in the evaluations had received prior training; in this study, the correlation coefficient of intragroup reliability was found to exceed 0.80 on repeated assessments. Furthermore, none of the psychiatrists were involved in the design of this study.

### 2.4. Blood Samples and Measurements

All patients were required to collect blood samples from 6 a.m. to 9 a.m. the next day after fasting for one night, and the sent these blood samples to the hospital laboratory for testing at 11 a.m. The thyroid function parameters, including free triiodothyronine (FT3), free thyroxine (FT4), thyroid stimulating hormone (TSH), antithyroglobulin (TgAb), and thyroid peroxidase antibody (TPOAb), were evaluated using a chemiluminescent microparticle immunoassay (CMIA) on an automated clinical ARCHITECT Immune 2000 SR analyzer (Abbott, Longford, Ireland). Additionally, fasting blood glucose (FBG) and lipid profile, including low density lipoprotein cholesterol (LDL-C), high density lipoprotein cholesterol (HDL-C), cholesterol (TC), and triglycerides (TG), were measured using an automated analyzer (ARCHITECT c8000 system, Abbott Laboratories, Illinois, USA).

### 2.5. Statistical Analysis

The first step was to compare the prevalence of suicide attempt between male and female MDD patients. The second step was to analyze the factors associated with suicide attempt among male and female patients separately. The data analysis was conducted using SPSS version 25.0 in this study.

First, the Kolmogorov–Smirnov test was used to determine the normality of data distribution. Continuous variables with normal distribution were presented as mean ± standard deviation (SD), and continuous data with non-normal distribution were logarithmically transformed into normal distribution and then presented as mean ± SD, while categorical variables were reported as frequency and percentage (%). Second, the chi-square test for categorical variables and the independent sample *t*-test for continuous variables were used to compare the differences between male and female groups. Third, in variables with homogeneous variance (Levene homogeneity test, *p* > 0.05), we employed 2 × 2 ANOVAs to examine the interaction between sex and suicide attempts, taking into account suicide attempts (two levels: yes and no) and sex (two levels: male and female). The purpose of this step was to compare the clinical parameters of MDD patients in the suicide attempt subgroup and the sex subgroup, as well as whether there is an interaction between these two subgroups. Further, independent sample *t*-tests, chi-square tests, and univariate analyses were performed separately for the male and female groups to assess clinical parameter differences subgroups with and without suicide attempts. Finally, multivariate binary logistic regression analysis was conducted with the presence of suicide attempts as the dependent variable (yes = 1, no = 0) and variables with *p* < 0.1 in univariate analysis as the independent variable, to identify the variables associated with suicide attempts in male and female patients. All *p*-values were two-tailed, with *p* < 0.05 indicating statistical differences. To account for multiple comparisons, the Bonferroni correction was used to adjust for multiple tests.

## 3. Results

### 3.1. Sex Differences in Demographic and Clinical Parameters in FEND Patients with MDD

A total of 1,718 FEDN MDD patients were enrolled, including 588 males and 1,130 females, of which 112 males (19.05%) and 234 females (20.71%) had suicide attempts.  Table 1 summarizes the demographic characteristics of patients and associated factors. Compared to male patients, female patients had a higher age (35.78 ± 12.45 vs. 33.12 ± 12.22, *p* < 0.001), an older onset age (35.57 ± 12.34 vs. 32.91 ± 12.09, *p* < 0.001), and more marriages (*χ*^2^ = 7.31, *p*=0.007 < 0.05), more psychotic symptoms (*χ*^2^ = 3.83, *p*=0.050), lower FT3 (4.88 ± 0.72 vs. 4.95 ± 0.73, *p*=0.047), and higher SBP (119.86 ± 11.16 vs. 118.76 ± 10.40, *p*=0.047). In terms of education, the majority of males had high school and undergraduate degrees, while females had middle school and graduate degrees (*χ*^2^ = 11.66, *p*=0.009).

### 3.2. Sex Differences in the Prevalence, Demographic, and Clinical Features in FEND Patients with MDD of Suicide Attempts

There was no difference in the percentage of male and female patients who had suicide attempts, with 19.0% for male patients and 20.7% for female patients (*χ*^2^ = 0.66, *p*=0.416). In Table 2, ANOVA was used to investigate the correlation between suicide attempts and sex. Suicide attempts (two levels, with and without suicide attempts) and sex (two levels, male and female) were used as intergroup variables for this two-way ANOVA. The results showed that suicide attempts had an effect on HAMD (*F* = 179.17, *p* < 0.001), TC (*F* = 93.57, *p* < 0.001), HDL-C (*F* = 32.46, *p* < 0.001), TG (*F* = 5.40, *p*=0.020 < 0.05), and LDL-C (*F* = 29.71, *p* < 0.001). There were also sex differences in age (*F* = 9.86, *p*=0.002) and age of onset (*F* = 10.09, *p*=0.002), as well as such differences between subgroups (age: *F* = 4.02, *p*=0.045; age of onset: *F* = 3.92, *p*=0.048), but there was no interaction between them (all *p* > 0.05). Suicide attempts also played a role in the course of the disease (*F* = 7.38, *p*=0.007). In addition, there were no clear SA × sex effects (all *p* > 0.05). Independent sample *t*-tests, chi-square tests, and univariate analysis were performed for both men and women.

### 3.3. The Related Factors of MDD with Suicide Attempts in Male FEDN Patients

In the male group, the number of MDD patients with SA and those without SA was 112 (19.0%) and 476 (81.0%), respectively. Patients with SA had higher HAMD scores (32.09 ± 3.11 vs. 29.86 ± 2.74) and HAMA scores (23.26 ± 3.41 vs. 20.05 ± 3.08), as well as higher TSH (6.51 ± 2.60 vs. 4.58 ± 2.30), Lg (TgAb) (1.69 ± 0.57 vs. 1.44 ± 0.47), Lg (TPOAb) (1.71 ± 0.65 vs. 1.33 ± 0.45), FBG (5.62 ± 0.68 vs. 5.31 ± 0.62), TC (5.78 ± 1.04 vs. 5.09 ± 1.02), LDC-L (3.24 ± 0.94 vs. 2.90 ± 0.82), SBP (122.33 ± 11.40 vs. 117.92 ± 9.98), and DBP (77.88 ± 8.32 vs. 75.43 ± 6.42), but lower HDL-C (1.14 ± 0.29 vs. 1.24 ± 0.30) (all *p* < 0.05). In addition, patients with SA had more severe anxiety symptoms (31.3% vs. 6.5%, *χ*^2^ = 55.68, *p* < 0.001) and psychotic symptoms (22.3% vs. 4.6%, *χ*^2^ = 38.62, *p* < 0.001).

Further, univariate analysis results (Table 3) showed that duration of diseases, HAMD and HAMA scores, TSH, Lg (TGAb), Lg (TPOAb), FBG, TC, HDL-C, LDL-C, BMI, SBP, DBP, severe anxiety, and psychotic symptoms were related to suicide attempts in the male group (all *p*  < 0.1). The multivariate logistic regression model (Table 4) revealed that the related factors of male patients with MDD accompanied by suicide attempts included HAMA score (OR = 1.28, 95% CI: 1.19–1.38, *p* < 0.001), Lg (TPOAB) (OR = 2.44, 95% CI: 1.60–3.73, *p* < 0.001), FBG (OR = 1.65, 95% CI: 1.15–2.39, *p*=0.007), TC (OR = 1.42, 95% CI: 1.13–1.80, *p*=0.003), and BMI (OR = 0.85, 95% CI: 0.75–0.96, *p*=0.008). The Cox and Snell *R*^2^ of this model is 0.202, and the Nagelkerke *R*^2^ is 0.324, with an accuracy of 84.7%.

### 3.4. The Related Factors of MDD with Suicide Attempts in Female FEDN Patients

In the female group, the number of people with SA and those without SA was 234 (20.7%) and 896 (79.3%), respectively. According to between-group comparisons, patients with SA had a longer course of disease (6.94 ± 4.76 vs. 6.18 ± 4.63), higher HAMD scores (32.31 ± 2.78 vs. 29.78 ± 2.75) and HAMA scores (23.75 ± 3.60 vs. 20.12 ± 3.08), higher TSH (6.81 ± 3.20 vs. 4.69 ± 2.28), Lg (TgAb) (1.72 ± 0.59 vs. 1.46 ± 0.47), Lg (TPOAb) (1.65 ± 0.65 vs. 1.36 ± 0.45), FBG (5.57 ± 0.77 vs. 5.37 ± 0.61), TC (5.77 ± 1.14 vs. 5.13 ± 1.09), TG (2.32 ± 1.00 vs. 2.14 ± 0.97), LDL-C (3.20 ± 0.89 vs. 2.94 ± 0.85), SBP (125.39 ± 12.13 vs. 118.42 ± 10.42), and DBP (78.89 ± 7.37 vs. 75.22 ± 6.26), while HDL-C was lower (1.14 ± 0.29 vs. 1.24 ± 0.28). These variations were all statistically significant (all *p* < 0.05). In addition, the differences in education level (*χ*^2^ = 9.40, *p*=0.024), severe anxiety symptoms (35.5% vs. 7.7%, *χ*^2^ = 122.90, *p* < 0.001), and psychotic symptoms (26.9% vs. 6.8%, *χ*^2^ = 76.85, *p* < 0.001) were also statistically significant.

Further, univariate analysis results (Table 3) showed that duration of diseases, HAMD and HAMA scores, TSH, Lg (TGAb), Lg (TPOAb), FBG, TC, HDL-C, TG, LDL-C, SBP, and DBP, as well as level of education and severe anxiety psychotic symptoms, was related factors with suicide attempts in the female group (all *p* < 0.1). The multivariate logistic regression model (Table 5) showed that HAMD score (OR = 1.13, 95% CI: 1.05–1.22, *p*=0.001), HAMA score (OR = 1.26, 95% CI: 1.18–1.34, *p* < 0.001), Lg (TPOAb) (OR = 2.00, 95% CI: 1.488–2.683), and SBP (OR = 1.04, 95% CI: 1.02–1.06, *p* < 0.001) were related factors for female MDD patients with SA. The Cox and Snell *R*^2^ of this model is 0.210, and the Nagelkerke *R*^2^ is 0.328, with an accuracy of 83.5%.

## 4. Discussion

To our knowledge, this is the first large-scale clinical study aimed to investigate sex differences in suicide attempts among FEDN patients with MDD. Our research findings revealed several important points: (1) there is no statistically significant difference in the rates of suicide attempts between males and females with MDD; (2) there are differences in sociodemographic information and clinical parameters between male and female patients with suicide attempts in FEDN MDD patients; and (3) there are sex differences in clinical variables related to suicide attempts in MDD patients.

### 4.1. Sex Differences in Suicide Attempt Rates among MDD Patients

In our study, the number of male and female patients was 588 and 1,130, respectively (with a ratio of 1 : 2), which was consistent with most previous studies that have confirmed that women were more likely to suffer from depression, about twice as many as men [33, 34]. However, our research showed that the rate of suicide attempts was similar for both men and women, with 19% of men versus 20.7%. Although the rate among females was slightly higher, this difference was not statistically significant. This aligns with a study conducted in the Netherlands [35] that also found no significant sex differences in the rate of suicide attempts among MDD patients. It is important to note that previous research on sex differences in suicide attempts among MDD patients has yielded conflicting results. For example, Schaffer et al. [24] discovered that being female was independently associated with an increase in the incidence and intensity of suicidal ideation among patients with a current major depressive episode. Conversely, Brådvik et al. [16] found that men faced a high risk of long-term suicide. In addition, Chen et al.'s [36] study showed that although repeated suicide attempts were more common in males, the risk of exacerbating suicide was more pronounced in females. Moreover, different age groups also exhibit sex differences in suicide attempts among MDD patients. Lewinsohn et al. [37] reported that female adolescents exhibited significantly higher rates of suicide attempts compared to male adolescents, but this sex difference disappeared by the age of 19. However, Beghi et al. [38] found that male sex played a more significant role in predicting completed suicides in elderly patients. Additionally, the sex differences in suicide attempts among MDD patients are influenced by race. An analysis [39] of the 2018 NSDUH data in the United States showed an increased risk of suicide attempts among Hispanic women, black men, and individuals from other racial backgrounds. The differences in datasets and evaluation methods used in previous studies may contribute to these inconsistent findings. Additionally, our study population only included MDD patients experiencing their first episode and with drug naïve. Therefore, we could compare the rates of suicide attempts by sex at the age group level in subsequent data analysis. To obtain a more comprehensive understanding, it would be beneficial to conduct similar surveys using diverse samples across different regions and ethnicities.

### 4.2. Sex Differences in Demographic Characteristics and Clinical Parameters of MDD Patients with or without Suicide Attempts

We also found that the levels of TSH, TGAb, and TPOAb were relatively higher in both male and female patients with suicide attempts. This finding aligned with previous research conducted by Shen et al. [40], which suggested a certain correlation between thyroid function indicators and suicide in MDD patients [40]. Furthermore, our study revealed that patients with suicide attempts had higher levels of TC and LDL-C, while their HDL-C levels were lower. This corresponded to the conclusions in a previous study [41] on young MDD patients. These findings suggested that dyslipidemia, characterized by elevated TC and LDL-C levels and reduced HDL-C levels, might be associated with an increased risk of suicide attempts in MDD patients. Interestingly, we observed a statistically significant difference in TG levels in the female group, but not in the male group. The relevant research has been extensively reported and controversial. For example, some researchers reported higher levels of TG, TC, and LDL-C in patients with suicidal depression, as seen in the study conducted by Koponen et al. [42]. However, other studies have indicated a relationship between recent suicide attempts or status, low serum TG levels, and high HDL in MDD patients [43]. Interestingly, Bartoli et al. [44] in their study found no association between serum cholesterol levels and suicide attempts. These conflicting results can be attributed to various factors, including age, disease duration, comorbidities, drug use, or lifestyle choices among the participants. Therefore, we carefully selected MDD patients who were first-episode and drug naïve, and we additionally stratified the data by sex to minimize the impact of an uneven distribution of male and female patients on the results. By employing these measures, we aimed to minimize the influence of these factors and provide a clearer understanding of the relationship between thyroid function indicators, blood lipids, and suicide attempts in MDD patients. Moving forward, we plan to further examine whether the above findings are consistently present in various types of MDD patients, such as those with psychotic symptoms, anxiety symptoms, obesity, and suicide attempts within 2 weeks. The ultimate goal of our research is to contribute to personalized medication in clinical diagnosis and treatment for MDD patients, while also minimizing the potential side effects of drugs on thyroid function and blood lipids. Ultimately, we hope that by addressing these factors, we can effectively reduce suicide rates among individuals with MDD.

Interestingly, our study found that, unlike the male group, the female group exhibited statistically significant differences in disease progression and education level when comparing patients with and without SA. It was particularly noteworthy that female patients who had received secondary and higher education were less likely to commit suicide compared to those who had only received primary education. This association, however, was not observed in male patients. Previous studies have found that individuals without a college degree are more likely to report suicidal ideation compared to those with higher levels of education [39]. This suggested that higher levels of education may serve as a protective factor against suicidal ideation, possibly because individuals with higher education levels have a better understanding of mental health issues like depression and suicide, as well as a greater propensity to seek help. Furthermore, our study also found that female patients with higher levels of education had fewer suicide attempts. This could be attributed to the fact that women generally possess a greater awareness and knowledge of depression compared to men, which allows them to recognize and openly discuss their depressive symptoms [45]. This awareness may enable women to seek help and support, reducing the likelihood of attempting suicide. Moreover, societal attitudes toward mental health may contribute to the lower rate of suicide attempts among educated females. Women often face less stigmatization when it comes to mental health, meaning they are more likely to receive understanding and empathy from their social circles. This supportive environment can potentially serve as a protective factor against suicide attempts [45]. In addition, our analysis found that MDD patients with psychiatric and severe anxiety symptoms were 5–6 times more prone to have suicide attempts than those without these symptoms in both males and females. Similar results have been observed in other recent studies that demonstrated the elevated risk of suicide attempts among depressed individuals with comorbid anxiety disorders or severe psychotic symptoms [46, 47]. However, the multivariate analysis did not reveal any significant correlations between the aforementioned factors. This outcome could be attributed to the complex interplay between individual components, suggesting that the relationship between education level, sex, psychotic symptoms, and suicidal behavior involves intricate mechanisms that are not yet fully understood. It is also possible that confounding variables, not accounted for in this study, may influence the observed associations. In order to gain a more comprehensive understanding of these findings, further analysis is necessary. Additional research should aim to identify the underlying reasons behind the observed relationships and validate the study's results using different datasets.

### 4.3. Sex Differences in Factors Related to Suicide Attempts in MDD Patients

Another important finding from our study was that the clinical factors associated with suicide attempts exhibited sex-specific variations. In male patients, the five clinical variables, namely HAMA, Lg (TPOAb), FBG, TC, and BMI were significantly correlated with suicide attempts. Conversely, among female patients, HAMA, HAMD, Lg (TPOAb), and SBP were identified as predictors of suicide attempts. Notably, Lg (TPOAb) emerged as a consistent predictor of suicide attempts in both male and female groups. This observation aligned with previous studies demonstrating elevated TPOAb levels in individuals with a history of suicide attempts compared to those without such history [48]. Some studies proposed that elevated TPOAb levels may indicate the presence of autoimmune diseases, potentially impacting the uric acid pathway and overactivating the HPA axis (hypothalamic–pituitary–adrenal). Additionally, increased inflammatory mediator levels may disrupt monoamine metabolism, ultimately contributing to severe emotional and behavioral disorders and heightening susceptibility to suicide [49]. Furthermore, our investigation revealed a positive association between HAMA, a marker of anxiety, and suicide attempts in both male and female MDD patients. Interestingly, prior research data indicated that individuals without a history of suicide attempts exhibited higher levels of overall anxiety compared to those with suicide attempts [50, 51], underscoring the potential role of heightened anxiety in instigating a fear of mortality. A comprehensive meta-analysis by Bentley et al. [52] supported anxiety as a predictive factor for suicidal ideation and attempts, rather than completed suicide. This emphasized the nuanced impact of anxiety on distinct suicide outcomes and underscored the need for outcome-specific considerations in the following research. Notably, our study focused on MDD patients with suicide attempts, extending beyond individuals solely with anxiety disorders, which may contribute to the observed outcome disparities. These findings have not only paved the way for novel avenues of inquiry into the biological underpinnings of suicidal ideation, attempts, and completed suicide in MDD patients but also hold promise for enhancing the precision and individualization of clinical interventions. Additionally, our investigation unveiled elevated levels of TC in both male and female MDD patients with suicide attempts compared to those without such history, with a significant positive correlation among male patients. TC emerged as a persistent risk factor for suicide attempts across varying levels [53, 54, 55], suggesting its potential utility in long-term risk assessment. Discrepancies in findings across studies may stem from differences in sample characteristics and clinical contexts. Our study enriched the understanding of these factors at a clinical level and underscored the imperative of identifying and addressing these factors in MDD patients with suicidal tendencies.

In female patients, positive correlations were observed between SBP and HAMD scores with suicide attempts. However, previous research presented conflicting viewpoints on this association. Some scholars have reported no significant link between SBP and depressive symptoms in both sexes [56]. Notably, their study did not establish a connection between blood pressure and suicide attempts, focusing on data derived from an elderly cohort, which may account for the disparities. Scalco et al. [57] proposed a reciprocal relationship between hypertension and depression, highlighting higher prevalence rates of hypertension in individuals with depression, and vice versa, perpetuating a cyclical pattern. Similarly, research on patients with coronary heart disease and hypertension indicated that those with higher levels of depression had higher suicidal ideation [58]. Conversely, recent findings suggested that adverse changes in vascular wall stiffness (VWS) and central blood pressure (CBP) were more pronounced in men with depression compared to women [59]. Multiple factors may contribute to the variability in research outcomes, including disparities in physical fitness across different racial groups, variations in criteria for defining depressive symptoms, and the specific focus of our study on drug naïve, first-episode MDD patients. Moreover, the severity of depression was closely related to an increased risk of suicide [60, 61]. A Danish cohort study also found that the more severe the first episode of MDD, the more severe the long-term trajectory, especially in female patients [62]. However, sex-specific differences in factors associated with suicide attempts among MDD patients remain inconclusive. Considering that our study population is FEND patients with MDD, while other studies involve MDD or depression patients, our findings need to be replicated in a larger population.

Our study also found a negative correlation between BMI and suicide attempt in male patients. Numerous studies have reported the relationship between obesity, depression, and suicide attempts. While some studies have reported no correlation between obesity and depression [63, 64], others have suggested that obese women were more likely to experience depressive symptoms and suicide attempts than men [65, 66]. A recent European study further demonstrated a significant correlation between elevated BMI and increased suicidal tendencies [67]. However, there is conflicting evidence regarding the impact of high BMI on depression and suicide. For example, a study from China [68] reported that obesity may actually reduce the incidence of depression in elderly men. Similarly, another study indicated an inverse relationship between BMI and the risk of complete suicide, particularly among males [69]. However, among women, high BMI was associated with an increased risk of attempted suicide but a reduced probability of complete suicide. This indicated that the relationship between BMI and suicide may be influenced by the specific outcomes of suicide. Perera et al. [70] also pointed out variations in the association between BMI and complete suicide versus attempted suicide. Like other studies, we also found a negative correlation between BMI and male suicide attempts, but we did not find a statistically significant difference in BMI between the SA group and the non-SA group. We speculate that there may be several reasons for this result: first, according to the guidelines of the China Obesity Working Group, there were fewer cases of obesity (≥28 kg/m^2^) in our data (28 males and 36 females). Second, our subjective definition of suicide attempts may have led to underestimations due to sensitivity biases. Third, body weight perception (BWP) has been linked to suicidal ideation, with women perceiving themselves as overweight being more prone to such thoughts [71]. Fourth, men's pursuit of muscle development may contribute to higher BMI and better physical fitness, which can alleviate symptoms of depression. Lastly, various confounding factors, such as ecological and social status, personal personality traits, and living environment, may influence the relationship between BMI and suicide, but we did not collect them. Therefore, further data collection is warranted to better explore the interplay of biological sex, physiological factors, and psychological factors in the relationship between obesity, depressive symptoms, and suicide attempts.

In the male group, we also discovered that FBG was correlated with suicide attempts. Previous research has substantiated the association between elevated FBG levels and suicide. For example, investigations have demonstrated that individuals with depression who have suicide attempts exhibit higher blood glucose levels during baseline assessments and 2-hr oral glucose tolerance tests (OGTT) [42]. Similarly, a longitudinal study conducted over a span of 14 years revealed that heightened fasting blood sugar levels elevate the risk of suicide [72]. Additionally, Ko et al. [73] observed that higher FBG levels may increase the risk of suicide. Given that glucose is indispensable for the energy requirements of brain cells, certain cognitive processes, such as self-control and emotion regulation, are intricately linked to the availability of glucose within brain cells [74]. In light of these considerations, we propose a hypothesis suggesting that alterations in blood sugar levels may impact the absorption of glucose by brain cells, consequently leading to suicide attempts. However, the above findings have yet to be explored in terms of sex differences, and further clinical validation is necessary to comprehend the biological disparities in suicide attempt risk variables between sexes.

### 4.4. Limitations

Several limitations should be acknowledged in our study. First, as this was a cross-sectional study, we were unable to investigate the direct causal association between various factors and suicide attempts. Future research should adopt a longitudinal study design to more comprehensively explore these associations. Second, our sample was derived exclusively from the psychiatric outpatient department of a general hospital in Taiyuan City, Shanxi Province, China, and solely consisted of individuals from the Han population. Consequently, the generalizability of our findings to all patients with depression is limited, necessitating the replication of our results in diverse populations encompassing various racial backgrounds and clinical contexts. Third, systematic evaluation techniques were not employed to assess suicidal behavior or ideation in our patients. In addition, we did not obtain precise information regarding the severity, modality, real threat, plans, ideation, and temporal dimensions of suicide attempts. These factors hold significance when examining suicide risk, as an attempt made recently may exhibit distinct scale scores and biochemical indicators compared to an attempt made 1 month ago. Given that parameters such as blood pressure, FBG, thyroid function indicators, and BMI are not static, the temporal aspect assumes importance. Therefore, in future studies, specific suicide questionnaires should be used to collect information related to suicide attempts. Additionally, it is imperative to consider the postdiagnosis trajectory of these patients and collect relevant data, such as suicide attempt rates 1 year, 3 years, or even over a lifetime following the initial diagnosis. Fourth, the sample comprised more females, accounting for 65.77% of the study participants meaning that the results might not be generalizable to males and should be interpreted with caution. Finally, several confounding factors that potentially exert an impact on the sex disparities in suicide attempts among MDD patients, such as smoking habits, personality traits, socioeconomic status, and biological factors, were not assessed. Subsequent investigations should account for these additional confounders to comprehensively examine sex differences in suicide attempts among individuals with MDD.

## 5. Conclusion

In summary, our study yielded several noteworthy findings. First, we observed no sex difference in the rates of suicide attempts among FEND patients with MDD. Second, we found that individuals, regardless of sex, who had suicide attempts exhibited higher LDL-C levels and lower HDL-C levels. This suggested that we need to pay attention to the level of blood lipids in the clinical treatment of depression, especially in the population with the possibility of coronary atherosclerotic disease. Third, Lg (TPOAb) and severe anxiety symptoms were positively correlated with suicide attempts in both male and female groups, providing us with a basis for thyroid function testing and HAMA assessment in MDD patients. Fourth, among female patients, there were significant differences in disease course, education level, and TG level between the suicide attempts group and the nonsuicide attempts group, whereas no such differences were observed in the male group. Furthermore, severe depressive symptoms and systolic blood pressure were positively correlated with suicide attempts in female patients. In male patients, TC and FBG values exhibited positive correlations with suicide attempts, while BMI displayed a negative correlation. Therefore, in the identification and prevention of suicide in MDD patients, we can conduct targeted examinations and evaluations for male and female patients. Collectively, our study enhances our understanding of sex disparities in suicide attempts and provides valuable insights for more effective prediction and prevention strategies.

## Figures and Tables

**Table 1:** Sex difference: sociodemographic and clinical characteristics among FEDN patients with MDD.

Characteristic	Total (*n* = 1,718)	Male (*n* = 588)	Female (*n* = 1,130)	*t*	*p*
Age (years)	34.87 ± 12.43	33.12 ± 12.22	35.78 ± 12.45	−4.22	<0.001
Duration of disease (months)	6.31 ± 4.73	6.25 ± 4.86	6.34 ± 4.66	−0.39	0.700
Age of onset (years)	34.66 ± 12.31	32.91 ± 12.09	35.57 ± 12.34	−4.26	<0.001
HAMD	30.30 ± 2.94	30.28 ± 2.95	30.30 ± 2.94	−0.15	0.883
HAMA	20.80 ± 3.47	20.66 ± 3.38	20.87 ± 3.52	−1.20	0.231
TSH (uIU/ml)	5.07 ± 2.56	4.95 ± 2.48	5.13 ± 2.60	−1.42	0.157
Lg (TGAb) (IU/l)	1.49 ± 0.50	1.49 ± 0.50	1.51 ± 0.51	−0.89	0.373
Lg (TPOAb) (IU/l)	1.41 ± 0.51	1.41 ± 0.51	1.42 ± 0.51	−0.43	0.666
FT3 (pmol/l)	4.90 ± 0.72	4.95 ± 0.73	4.88 ± 0.72	1.99	0.047
FT4 (pmol/l)	16.70 ± 3.10	16.72 ± 3.15	16.69 ± 3.07	0.20	0.838
FBG (mmol/l)	5.40 ± 0.65	5.37 ± 0.64	5.41 ± 0.65	−1.20	0.229
TC (mmol/l)	5.25 ± 1.11	5.22 ± 1.06	5.26 ± 1.13	−0.79	0.429
HDL-C (mmol/l)	1.22 ± 0.29	1.22 ± 0.30	1.22 ± 0.28	0.48	0.629
TG (mmol/l)	2.17 ± 0.99	2.14 ± 1.00	2.18 ± 0.98	−0.68	0.497
LDL-C (mmol/l)	2.98 ± 0.86	2.96 ± 0.85	3.00 ± 0.87	−0.77	0.441
BMI (kg/m^2^)	24.37 ± 1.92	24.41 ± 2.04	24.35 ± 1.86	0.62	0.539
SBP (mmHg)	119.48 ± 10.91	118.76 ± 10.40	119.86 ± 11.16	−1.99	0.047
DBP (mmHg)	75.95 ± 6.74	75.90 ± 6.89	75.98 ± 6.67	−0.23	0.820
	Total (*n* = 1,718)	Male (*n* = 588)	Female (*n* = 1,130)	*χ* ^2^	*p*-Value
Suicide attempters (*n*, %)	—	—	—	0.66	0.416
Yes	346 (20.1)	112 (19.0)	234 (20.7)	—	—
No	1,372 (79.9)	476 (81.0)	896 (79.3)	—	—
Marital status (*n*, %)	—	—	—	7.31	0.007
Marriage	1,216 (70.8)	392 (66.7)	824 (72.9)	—	—
Single	502 (29.2)	196 (33.3)	306 (27.1)	—	—
Psychotic symptoms (*n*, %)	—	—	—	3.83	0.050
Yes	171 (10.0)	47 (8.0)	124 (11.0)	—	—
No	1,547 (90.0)	541 (92.0)	1,006 (89.0)	—	—
Severe anxiety symptoms (*n*, %)	—	—	—	1.73	0.188
Yes	218 (12.7)	66 (11.2)	152 (13.5)	—	—
No	1,500 (87.3)	522 (88.8)	978 (86.5)	—	—
Education (*n*, %)	—	—	—	11.66	0.009
Junior	413 (24.0)	125 (21.3)	288 (25.5)	—	—
Senior	760 (44.2)	271 (46.1)	489 (43.3)	—	—
College	449 (26.1)	170 (28.9)	279 (24.7)	—	—
Postgraduate	96 (5.6)	22 (3.7)	74 (6.5)	—	—

*Note*: HAMD, 17-item Hamilton Rating Scale for Depression; HAMA, 14-item Hamilton Anxiety Rating Scale; TSH, thyroid-stimulating hormone; TGAb, thyroglobulin antibody; TPOAb, thyroid peroxidase antibody; FT3, free triiodothyronine; FT4, free thyroxine; FBG, fasting blood glucose; TC, total cholesterol; HDL-C, high-density lipoprotein cholesterol; TG, triglyceride; LDL-C, low-density lipoprotein cholesterol; BMI, body mass index; SBP, systolic pressure; DBP, diastolic pressure.

**Table 2:** Demographic and clinical characteristics between MDD patients with and without suicide attempts, grouped by sex.

Characteristic	With suicide		Without suicide		Sex *F* (*p*-value)	Group *F* (*p*-value)	Sex × subgroup *F* (*p*-value)
	Male	Female	Male	Female			
Age (years)	34.60 ± 12.68	36.85 ± 12.15	32.78 ± 12.10	35.50 ± 12.51	9.86 (0.002)	4.02 (0.045)	0.09 (0.767)
Duration of disease (months)	6.96 ± 5.16	6.94 ± 4.76	6.08 ± 4.78	6.18 ± 4.63	0.02 (0.892)	7.38 (0.007)	0.04 (0.835)
Age of onset	34.36 ± 12.60	36.62 ± 12.06	32.58 ± 11.95	35.30 ± 12.40	10.09 (0.002)	3.92 (0.048)	0.09 (0.771)
HAMD scores	32.09 ± 3.11	32.31 ± 2.78	29.86 ± 2.74	29.78 ± 2.75	0.17 (0.682)	179.17 (<0.001)	0.71 (0.400)
FT3 (pmol/l)	4.88 ± 0.74	4.93 ± 0.72	4.97 ± 0.72	4.87 ± 0.72	0.31 (0.578)	0.09 (0.770)	2.85 (0.092)
FT4 (pmol/l)	16.80 ± 3.16	16.60 ± 3.12	16.71 ± 3.15	16.71 ± 3.06	0.20 (0.651)	0.00 (0.999)	0.23 (0.630)
TC (mmol/l)	5.78 ± 1.04	5.77 ± 1.14	5.09 ± 1.02	5.13 ± 1.09	0.07 (0.789)	93.57 (<0.001)	0.13 (0.719)
HDL-C (mmol/l)	1.14 ± 0.29	1.14 ± 0.289	1.24 ± 0.30	1.24 ± 0.28	0.06 (0.801)	32.46 (<0.001)	0.01 (0.946)
TG (mmol/l)	2.24 ± 1.04	2.32 ± 1.00	2.12 ± 0.99	2.14 ± 0.97	0.66 (0.418)	5.40 (0.020)	0.26 (0.608)
LDL-C (mmol/l)	3.24 ± 0.94	3.20 ± 0.89	2.90 ± 0.82	2.94 ± 0.85	0.00 (0.982)	29.71 (<0.001)	0.70 (0.404)

*Note*: HAMD, 17-item Hamilton Rating Scale for Depression; FT3, free triiodothyronine; FT4, free thyroxine; TC, total cholesterol; HDL-C, high-density lipoprotein cholesterol; TG, triglyceride; LDL-C, low-density lipoprotein cholesterol.

**Table 3:** Univariate analysis for suicide attempts in males and females with FEDN MDD.

Variables	Male (*n* = 588)		Female (*n* = 1,130)	
	OR (95% CI)	*p*	OR (95% CI)	*p*
Age (years)	1.01 (1.00, 1.03)	0.156	1.01 (1.00, 1.02)	0.139
Duration of disease (months)	1.04 (1.00, 1.08)	0.085	1.03 (1.00, 1.06)	0.027
Age of onset (years)	1.01 (1.00, 1.03)	0.161	1.01 (1.00, 1.02)	0.144
HAMD	1.31 (1.21, 1.41)	<0.001	1.39 (1.31, 1.48)	<0.001
HAMA	1.34 (1.25, 1.43)	<0.001	1.37 (1.31, 1.44)	<0.001
TSH (uIU/ml)	1.40 (1.27, 1.54)	<0.001	1.38 (1.30, 1.46)	<0.001
Lg (TGAb) (IU/l)	2.37 (1.64, 3.44)	<0.001	2.49 (1.92, 3.23)	<0.001
Lg (TPOAb) (IU/l)	3.46 (2.39, 5.01)	<0.001	2.69 (2.08, 3.48)	<0.001
FT3 (pmol/l)	0.84 (0.63, 1.12)	0.229	1.13 (0.93, 1.38)	0.223
FT4 (pmol/l)	1.01 (0.95, 1.08)	0.771	0.99 (0.94, 1.04)	0.672
FBG (mmol/l)	2.04 (1.48, 2.80)	<0.001	1.60 (1.29, 1.99)	<0.001
TC (mmol/l)	1.87 (1.52, 2.30)	<0.001	1.67 (1.46, 1.91)	<0.001
HDL-C (mmol/l)	0.30 (0.14, 0.60))	0.001	0.27 (0.16, 0.46)	<0.001
TG (mmol/l)	1.12 (0.92, 1.36)	0.276	1.20 (1.04, 1.38)	0.013
LDL-C (mmol/l)	1.61 (1.26, 2.06)	<0.001	1.39 (1.18, 1.64)	<0.001
BMI (kg/m^2^)	0.89 (0.80, 0.99)	0.031	1.04 (0.96, 1.13)	0.299
SBP (mmHg)	1.04 (1.02, 1.07)	<0.001	1.06 (1.05, 1.08)	<0.001
DBP (mmHg)	1.05 (1.02, 1.08)	0.001	1.09 (1.06, 1.11)	<0.001
Education	—	—	—	—
Junior	1.00 (ref)	—	1.00 (ref)	—
Senior	0.93 (0.54, 1.60)	0.790	0.65 (0.46, 0.91)	0.014
College	1.01 (0.57, 1.82)	0.964	0.58 (0.39, 0.87)	0.009
Postgraduate	1.58 (0.56, 4.46)	0.389	0.90 (0.50, 1.62)	0.718
Marriage status	—	—	—	—
Single	1.00 (ref)	—	1.00 (ref))	—
Marriage	1.47 (0.93, 2.32)	0.104	0.96 (0.69, 1.32)	0.787
Severe anxiety	—	—	—	—
No	1.00 (ref)	—	1.00 (ref)	—
Yes	6.53 (3.80, 11.20)	<0.001	6.59 (4.58, 9.48)	<0.001
Psychotic symptoms	—	—	—	—
No	1.00 (ref)	—	1.00 (ref)	—
Yes	5.93 (3.20, 12.99)	<0.001	5.04 (3.42, 7.44)	<0.001

*Note*: OR, odd ratio; CI, confidence interval; HAMD, 17-item Hamilton Rating Scale for Depression; HAMA, 14-item Hamilton Anxiety Rating Scale; TSH, thyroid-stimulating hormone; TGAb, thyroglobulin antibody; TPOAb, thyroid peroxidase antibody; FT3, free triiodothyronine; FT4, free thyroxine; FBG, fasting blood glucose; TC, total cholesterol; HDL-C, high-density lipoprotein cholesterol; TG, triglyceride; LDL-C, low-density lipoprotein cholesterol; BMI, body mass index; SBP, systolic pressure; DBP, diastolic pressure.

**Table 4:** Binary logistic regression analyses of determinants of suicide attempts in male FEND patients.

	*β*	Standard error	Wald	*p*-Value	95% confidence interval for OR		
					OR	Lower	Upper
Constant	−8.83	1.90	21.52	<0.001	0.00	—	—
HAMA scores	0.25	0.04	40.92	<0.001	1.28	1.19	1.38
Lg (TPOAb) (IU/l)	0.89	0.22	17.10	<0.001	2.44	1.60	3.73
FBG (mmol/l)	0.50	0.199	7.22	0.007	1.65	1.15	2.39
TC (mmol/l)	0.35	0.12	8.70	0.003	1.42	1.13	1.80
BMI (kg/m^2^)	−0.16	0.06	7.10	0.008	0.85	0.75	0.96

*Note*: OR, odd ratio; HAMA, 14-item Hamilton Anxiety Rating Scale; TPOAb, thyroid peroxidase antibody; FBG, fasting blood glucose; TC, total cholesterol; BMI, body mass index.

**Table 5:** Binary logistic regression analyses of determinants of suicide attempts in female FEND patients.

	*β*	Standard error	Wald	*p*-Value	95% confidence interval for OR		
					OR	Lower	Upper
Constant	−15.89	1.36	136.76	<0.001	0.00	—	—
HAMD scores	0.12	0.04	10.38	0.001	1.13	1.05	1.22
HAMA scores	0.23	0.03	53.97	<0.001	1.26	1.18	1.34
Lg (TPOAb) (IU/l)	0.69	0.15	21.21	<0.001	2.00	1.49	2.68
SBP (mmHg)	0.04	0.01	21.48	<0.001	1.04	1.02	1.06

*Note*: OR, odd ratio; HAMD, 17-item Hamilton Rating Scale for Depression; HAMA, 14-item Hamilton Anxiety Rating Scale; TPOAb, thyroid peroxidase antibody; SBP, systolic pressure.

## Data Availability

The datasets used and/or analyzed during the current study are available from the corresponding author upon reasonable request.
